# A novel biomimetic nanoplasmonic sensor for rapid and accurate evaluation of checkpoint inhibitor immunotherapy

**DOI:** 10.1007/s00216-024-05398-3

**Published:** 2024-06-20

**Authors:** Razia Batool, Maria Soler, Rukmani Singh, Laura M. Lechuga

**Affiliations:** Nanobiosensors and Bioanalytical Applications Group (NanoB2A), Catalan Institute of Nanoscience and Nanotechnology (ICN2), CSIC, BIST and CIBER-BBN, 08193 Bellaterra, Barcelona, Spain

**Keywords:** Localized surface plasmon resonance, Label-free analysis, Sensor biofunctionalization, Artificial cell membrane, Checkpoint inhibitors, Cancer immunotherapy

## Abstract

Immune checkpoint inhibitors (ICIs) emerged as promising immunotherapies for cancer treatment, harnessing the patient’s immune system to fight and eliminate tumor cells. However, despite their potential and proven efficacies, checkpoint inhibitors still face important challenges such as the tumor heterogeneity and resistance mechanisms, and the complex in vitro testing, which limits their widespread applicability and implementation to treat cancer. To address these challenges, we propose a novel analytical technique utilizing biomimetic label-free nanoplasmonic biosensors for rapid and reliable screening and evaluation of checkpoint inhibitors. We have designed and fabricated a low-density nanostructured plasmonic sensor based on gold nanodisks that enables the direct formation of a functional supported lipid bilayer, which acts as an artificial cell membrane for tumor ligand immobilization. With this biomimetic scaffold, our biosensing approach provides real-time, highly sensitive analysis of immune checkpoint pathways and direct assessment of the blocking effects of monoclonal antibodies in less than 20 min/test. We demonstrate the accuracy of our biomimetic sensor for the study of the programmed cell death protein 1 (PD1) checkpoint pathway, achieving a limit of detection of 6.7 ng/mL for direct PD1/PD-L1 interaction monitoring. Besides, we have performed dose–response inhibition curves for an anti-PD1 monoclonal antibody, obtaining a half maximal inhibitory concentration (IC_50_) of 0.43 nM, within the same range than those obtained with conventional techniques. Our biomimetic sensor platform combines the potential of plasmonic technologies for rapid label-free analysis with the reliability of cell-based assay in terms of ligand mobility. The biosensor is integrated in a compact user-friendly device for the straightforward implementation in biomedical and pharmaceutical laboratories.

**Graphical Abstract:**

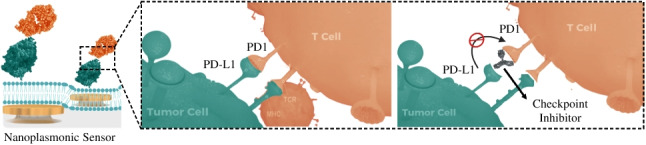

**Supplementary Information:**

The online version contains supplementary material available at 10.1007/s00216-024-05398-3.

## Introduction

Immune checkpoint inhibitors (ICIs) are a type of immunotherapy designed to counterbalance the tumor immunosuppressive environment, thereby harnessing and aiding the patient’s immune system to fight cancer. These drugs work by targeting specific cell receptors (i.e., immune checkpoints) that act as brakes on the immune system, preventing it from attacking cancer cells. By blocking these checkpoints, ICIs unleash the immune system to recognize and destroy tumor cells more effectively. One of the key immune checkpoints in cancer treatment is the programmed cell death 1 (PD1) receptor, mainly expressed on the surface of T cells. By upregulating its corresponding ligand (PD-L1), tumor cells are able to suppress the T cell’s killing effect, whereas the blockage of the PD1/PD-L1 pathway through ICIs allows for recovering the T cells effector function for destroying the cancer cells [1]. To date, monoclonal antibodies (mAbs) targeting either PD1 (e.g., nivolumab, pembrolizumab, cemiplimab) or PD-L1 (e.g., atezolizumab, durvalumab, avelumab) have been approved by the US Food and Drug Administration (FDA) and the European Medicines Agency (EMA) for the treatment of different cancers [2]. The significance of ICIs in cancer therapy lies in their ability to induce durable responses in various types of cancer, including melanoma; lung, bladder, and breast cancers; lymphoma; and many others. Unlike traditional cancer treatments like chemotherapy, which directly target cancer cells, ICIs have the potential to provide long-lasting benefits by priming the immune system to recognize and eliminate cancer cells throughout the body. This has led to significant improvements in the overall survival and quality of life for patients with advanced or metastatic cancers. However, it is important to note that not all patients respond to ICIs, and research is ongoing to better understand why some individuals benefit more than others to develop strategies to enhance their effectiveness. A significant limitation of ICIs therapy is the development of tumor resistance mechanisms. Cancer cells can adapt and evade immune surveillance through various mechanisms, including upregulation of alternative immune checkpoints, alterations in antigen presentation, and recruitment of immunosuppressive cells. Understanding and overcoming these resistance mechanisms are critical for improving the long-term efficacy of ICIs. Furthermore, the assessment of ICIs efficacy can be complex and may require novel analytical techniques that enable the rapid screening of different inhibitor candidates and their dose–response curves, the study of new immune checkpoints, or the characterization of the mechanism of action and the patient’s immune response.

Current screening and evaluation methods for immune checkpoint inhibitors include enzyme-linked immunosorbent assays (ELISA), fluorescence-based technologies, biolayer interferometry (BLI), nuclear magnetic resonance (NMR) spectroscopy, isothermal titration calorimetry (ITC), microscale thermophoresis (MST), and surface plasmon resonance (SPR) biosensing [3]. However, these techniques generally require expensive instrumentation or labelling, they can be laborious and time-consuming, and do not consider the mobility freedom or restrictions of the ligands when expressed on the cell membranes. To that, cell-based assays can be carried out using bioluminescence or fluorescent reporters in cell culture experiments, evaluating the activation of T cells by targeting specific receptors or cytokine secretion events [3]. Although these methods are appropriate to evaluate the basic biological functions and effects of ICIs, the results are often qualitative and can be biased due to labelling interferences or cell population heterogeneity. Moreover, cell culture experiments are also complex and tedious, requiring highly specialized personnel and dedicated infrastructure.

**Fig. 1:**
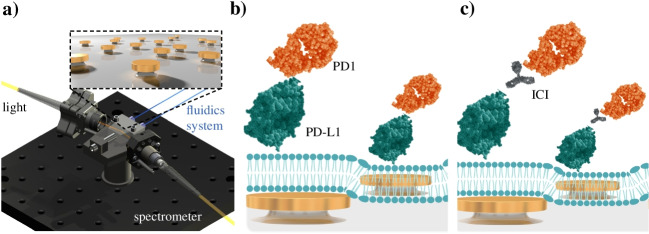
(**a**) Schematic illustration of LSPR biosensor device; (**b**) illustration of the biomimetic LSPR sensor approach for monitoring direction interaction of PD1 receptor with PD-L1 ligand; and (**c**) illustration of the immune checkpoint inhibitor (ICI) screening assay for PD1/PD-L1 pathway blocking. The figure is only for illustrative purposes; elements (nanodisks, lipids, proteins, and antibodies) are not to scale.

We propose the use of a biomimetic nanoplasmonic biosensor for the rapid screening and assessment of ICIs in a label-free and real-time format (Fig. 1), combining the major benefits of optical biosensor technologies and cell-based assays to deliver accurate and reliable data while minimizing the time-consuming culture procedures and possible labelling interferences. Our biosensor is based on the localized surface plasmon resonance (LSPR) phenomenon of metallic nanostructures, which allows for highly sensitive evanescence-field sensing of biomolecular interactions — similar to conventional SPR technology, and the integration in a compact device with user-friendly operation [4–6]. Besides, the nanoplasmonic sensor is coated with an artificial cell membrane that can be specifically functionalized with the immune checkpoint ligands, mimicking a tumor membrane in terms of molecular mobility, and facilitating the study of checkpoint receptors and/or inhibitors without the need of cell culture experiments. Plasmonic sensor systems with biomimetic assay properties have been previously reported for different biomedical applications, introducing important features and advantages for enhancing the analysis reliability and sensitivity [7]. Back in 2007, Jonsson et al. [8] described and fully characterized the formation of supported lipid bilayers (SLB) on nanoplasmonic sensors, demonstrating the lateral mobility of bioreceptors immobilized onto the lipid membrane as well as the enhancement of the protein binding to the cell membrane mimics, compared to conventional surface chemistry immobilization procedures. More recently, Yoon et al. [9] utilized a nanoplasmonic sensor for real-time visualization of the morphological changes of SLBs with applications in the design of antimicrobial surfaces. In terms of biomedical applications, plasmonic sensors with SLB have been demonstrated for the evaluation of T cell immunotherapies for cancer [10] and for monoclonal antibody therapy for COVID-19 [11]. In all these studies, however, the nanoplasmonic sensor surface is coated with a thin SiO_2_ layer (10–15 nm) in order to ensure a planar and highly hydrophilic substrate for the efficient formation of the SLB through spontaneous disruption of small unilamellar vesicles (SUVs). This coating strategy has shown to be useful, robust, and reliable, but it might entail sensitivity limitations when working with nanostructured LSPR sensors, exhibiting shorter penetration depths of the evanescent electromagnetic field than conventional SPR. To explain briefly, nanoplasmonic sensors formed by arrays of metallic nanoparticles (nanodisks, nanorods, etc.) exhibit highly localized resonances with a sensing interface reaching up to 50 nm [12]. This phenomenon has been exploited to increase the biosensor resolution and specificity, since the biomolecular recognition event occupies the majority of the evanescent field depth. However, the coating of the nanostructured sensor surface with a thin layer of SiO_2_ and the use of a SLB as immobilization scaffold (5–10 nm thick) [10] would push the recognition event away from the sensitive area of the sensor, drastically reducing the detection sensitivity.

In response to above challenges, we have designed and developed a low-density quasi-planar nanoplasmonic sensor that allows for the direct formation of SLB onto the sensing surface, eliminating the need for SiO_2_ coating and ensuring a high-sensitivity performance. By selecting the appropriate nanostructure geometry and density, we could fabricate a new LSPR sensor with comparable performance to the state-of-the-art technologies and a hydrophilic behaviour equivalent to bare glass. Our nanoplasmonic sensor can be fabricated and produced at large scales with cost-effective procedures, and it is integrated in a small device for decentralized and facile operation. We demonstrate the robust formation of artificial cell membranes directly onto the nanoplasmonic surface and its potential application for the label-free evaluation of ICIs as cancer immunotherapy. Particularly, we study the interaction of PD1 receptors with a PD-L1-functionalized lipid membrane (Fig. 1b), and the blocking effect and efficacy of anti-PD1 mAbs acting as checkpoint inhibitors (Fig. 1c). Due to its versatility, sensitivity, and rapid turnaround assay times (15–20 min/assay), this new biomimetic sensor platform could greatly contribute to the study, screening, and evaluation of alternative checkpoint inhibitors (e.g., antibodies, small molecules, aptamers), to investigate the immunosuppressive effects of different tumor ligands, or to explore additional immune checkpoints as candidates for novel therapeutic solutions.

## Materials and methods

### Chemical and biological reagents

Reagents for cleaning the substrates, sodium dodecyl sulphate 99% (SDS), and hydrogen peroxide (H_2_O_2_) 30%, were purchased from Sigma-Aldrich (Steinheim, Germany). Hydrochloric acid (HCl) 37%, sulfuric acid (H_2_SO_4_) 96%, acetone, and ethanol were provided by Panreac AppliChem (Barcelona, Spain). Lipids (POPC, 1-palmitoyl-2-oleoyl-sn-glycero-3-phosphocholine) and DOPS (1,2-dioleoyl-sn-glycero-3-phospho-L-serine) were acquired from Avanti Polar Lipids (Alabaster, AL, USA). All buffer compounds, including sodium acetate, Tween 20, sodium citrate dihydrate, tris (hydroxymethyl) amino-methane 99.8%, PBS (phosphate-buffered saline, pH 7.4), HEPES (N-(2-hydroxyethyl) piperazine-N-(2-ethanesulfonic acid), pH 7.24), and MES (2-(N-morpholino) ethane sulfonic acid, pH 5.0), as well as 1-ethyl-3-(3-dimethylaminopropyl) carbodiimide hydrochloride (EDC) and sulfo-N-hydroxysuccinimide (s-NHS) were obtained from Sigma-Aldrich (Steinheim, Germany). Programmed death-ligand 1 (PD-L1), programmed cell death protein 1 (PD1), and anti-PD1 monoclonal antibody (mAb) were purchased from GenScript (Leiden, The Netherlands).

### Numerical modeling and fabrication of nanoplasmonic sensors

Numerical analysis for the design and optimization of low-density short-ordered nanoplasmonic arrays of gold nanodisks (AuNDs: height 20 nm, diameters 80 and 100 nm) was performed using the finite element method (FEM)-based COMSOL Multiphysics. A three-dimensional unit cell of the structure (i.e., a single gold nanodisk on a SiO_2_ substrate) with cladding layer of water (refractive index 1.33) was utilized for the numerical analysis, as illustrated in Supplementary Information (SI) (Fig. S1a), considering periodic boundary condition in both lateral dimensions. P-polarized incident light with wavelengths ranging from 400 to 900 nm was employed for optical spectral analysis. Simulations were executed using the extra-fine meshing option provided by the analytical tool for improved precision. The substrate material, SiO_2_, was defined by a wavelength-dependent refractive index using the Sellmeier formula [13]. The refractive index of gold nanodisks (AuNDs) as a function of wavelength was implemented as an interpolation function in the COMSOL Multiphysics, with reference to [12]. The light input port was positioned at the bottom of the substrate, and light collection occurred through the output port after passing through the cladding layer. The frequency domain analysis was employed to observe the near-fields generated by the AuNDs nanoplasmonic arrays. The transmittance spectrum was measured for performance analysis, facilitating the design and optimization of dimensional parameters for the sensor.

For the nanofabrication of the designed AuNDs sensors (80 nm diameter, 20 nm height, 80 nm gap), we employed a procedure based on hole-mask colloidal lithography (HCL), which has been previously described [5, 6, 14]. Details are provided in the Supplementary Information (S2). The nanofabrication procedure renders short-ordered arrays of AuNDs with the desired dimensions onto SiO_2_ substrate and allows production at large scale and low cost.

### LSPR biosensor device description

The biosensor device employed is a proprietary technology, which is integrated into a compact platform (20 × 20 cm), and it has been described previously [5, 6, 14]. The device is based on a prism-coupling excitation scheme, operating at a fixed angle (*θ* = 70°). The nanoplasmonic sensors are placed in contact with a custom-made microfluidic device, which is connected to a continuous flow system provided by a syringe pump and including a sample injection valve with a 150-µL loop. The device monitors the binding events occurring onto the AuNDs sensors in real time by tracking the displacements of the LSPR resonance peak wavelength (Δ*λ*, nm).

### Formation of PD-L1-functionalized supported lipid bilayer (SLB)

Prior to forming a functional lipids membrane, the nanoplasmonic sensors underwent consecutive sonication cleaning at room temperature for 1 min in acetone, isopropanol, and Milli-Q water, respectively. Then, sensors were dried with N_2_ stream and placed in an UV/O_3_ Procleaner Plus (Bioforce Nanosciences, UT, USA) for 20 min, after which they were rinsed with ethanol and water and dried with N_2_ stream. The sensor chips were mounted on the LSPR setup, and a rapid in situ surface cleaning was performed by injecting 10 mM NaOH for 1 min to eliminate possible remaining residues on the sensor surface. We have previously demonstrated and characterized the successful formation of a stable lipid bilayer on SiO_2_-coated gold thin film using this identical cleaning procedure and the preparation of lipids vesicles followed our previously published strategy [10, 11]. In brief, a specific volume of lipid mixtures (POPC:DOPS, 10:1) was prepared in chloroform, dried overnight in a vacuum desiccator to remove residual solvent, and rehydrated using PBS (10 mM, pH 7.4) to form a lipid colloidal solution of 1 mg/mL. Vortexing and sonication resulted in a heterogeneous vesicle solution. These vesicles were then extruded through a 0.1-μm polycarbonate membrane using a mini extruder (Avanti Polar Lipids, Inc., Alabaster, AL, USA) to form small unilamellar vesicles (SUVs). SUVs solution was quickly injected onto the nanosensor surface, and the formation of the SLB was monitored in real time. A 10 mM NaOH solution was injected to ensure the stable formation of the lipid layer and to remove possible adsorbed vesicles. Once SLB was stabilized on the nanoplasmonic surface, PD-L1 Fc chimera molecules (50 µg/mL) were covalently immobilized to the reactive carboxylic groups (COOH) available from the DOPS phospholipids through EDC/NHS (0.4 M/0.1 M) chemistry in MES buffer (50 mM, pH 5). Ethanolamine (EA, 1 M, pH 8) was injected to deactivate all the unreacted carboxylic groups. All measurements were carried out with PBS as running buffer at 20 μL/min flow rate with 150 µL sample volume.

### Analysis of PD1/PD-L1 interaction

A PD1/PD-L1 binding curve was obtained by evaluating PD1 concentrations ranging from 7.6 to 5000 ng/mL diluted in HEPES buffer (50 mM, pH 7.4) at a flow rate of 20 µL/min. Following each concentration measurement, the interaction between PD1 and PD-L1 was disrupted by flushing with a 10 mM NaOH solution for 2 min at the same flow rate, and the biosurface was regenerated. Data analysis was conducted using GraphPad Prism 8 (GraphPad Software, CA, US) and Origin 8.0 (Origin Lab, MA, US). Calibration curves were constructed by plotting the mean sensor response (Δ*λ*) with its standard deviation (mean ± SD) against the target concentration. Data set fitting was performed using a one-site specific binding model curve. The limits of detection (LOD) and quantification (LOQ) were determined as the PD1 concentrations corresponding to three and ten times the standard deviation of the baseline sensor signal, respectively.

### Evaluation of anti-PD1 mAb as checkpoint inhibitors

A competitive assay was performed to evaluate the efficiency of anti-PD1 mAb as inhibitors of PD1/PD-L1 interaction. For that, PD1 samples at a fixed concentration (2000 ng/mL) were incubated for 10 min with anti-PD1 mAb across a range of doses from 0 to 6500 ng/mL. After the incubation, the samples were flowed over the PD-L1-biofunctionalized sensor and signals were acquired and normalized. Analyte concentrations were tested in triplicate, and the signals (mean ± SD) were plotted against the analyte concentration’s logarithmic value. An inhibitory dose–response fitting curve was used to fit the data and the half maximal inhibitory concentration (IC_50_) was calculated as the anti-PD1 mAb concentration corresponding to the 50% of the interaction inhibition signal.

## Results and discussion

### Design, fabrication, and characterization of low-density nanostructured plasmonic sensors

A major objective of this work was to develop a nanoplasmonic sensor that allows for direct formation of supported lipid membrane scaffolds while providing high sensitivity for label-free biomolecular analysis. To address this, our approach entails the design of a low-density nanostructured plasmonic sensor on a SiO_2_ substrate that may behave similar to a planar glass surface in terms of hydrophilicity, avoiding the additional SiO_2_ coating, as well as enabling the label-free refractometric sensing with maximum evanescent field penetration depths. In previous works, we demonstrated that short-ordered arrays of gold nanodisks (AuNDs, 100 nm diameter (*d*), 20 nm height (*h*)) exhibit highly sensitive LSPR in the visible region (*λ*_LSPR_ ~ 700 nm) [5, 6, 14]. Based on this premise and considering that nanostructures with a height of 20 nm behave as quasi-planar surfaces, we have designed a new nanoplasmonic sensor architecture by optimizing both the AuNDs diameter and the nanostructure gap. Through numerical simulations, we observed that by reducing the AuNDs to 80 nm, the LSPR peak could be tuned to shorter wavelengths within the visible region (*λ*_LSPR_ ~ 650 nm), narrowing the peak bandwidth and increasing the evanescent field penetration depth [15] (Fig. S1b). Then, the effect of the interspacing distance (gap, *g*) between two nanodisks, which is directly related to the density of nanodisks on the sensor, was analyzed (Fig. S1c and S1d). This study revealed that increasing the interspacing distance (from *g* = *d*/2 to *g* = 2*d*) yields a sharper peak (narrower FWHM) but with a slight decrease of peak intensity. The interspacing distance was selected equal to nanodisk’s diameter (*g* = *d* = 80 nm) as it provides optimum values for both the FWHM and peak intensity. However, the numerical analysis indicated that the interspacing distance could vary from *d* to 2*d* without significantly affecting the resonance peak position and intensity, providing a positive margin within fabrication tolerance [16]. Moreover, by choosing a larger gap size, we decrease the overall density of AuNDs on the sensor, which is advantageous for forming supported lipid bilayer (SLB). By using this theoretical interspacing gap, we could expect a gold nanodisk’s density of below 10%.

**Fig. 2:**
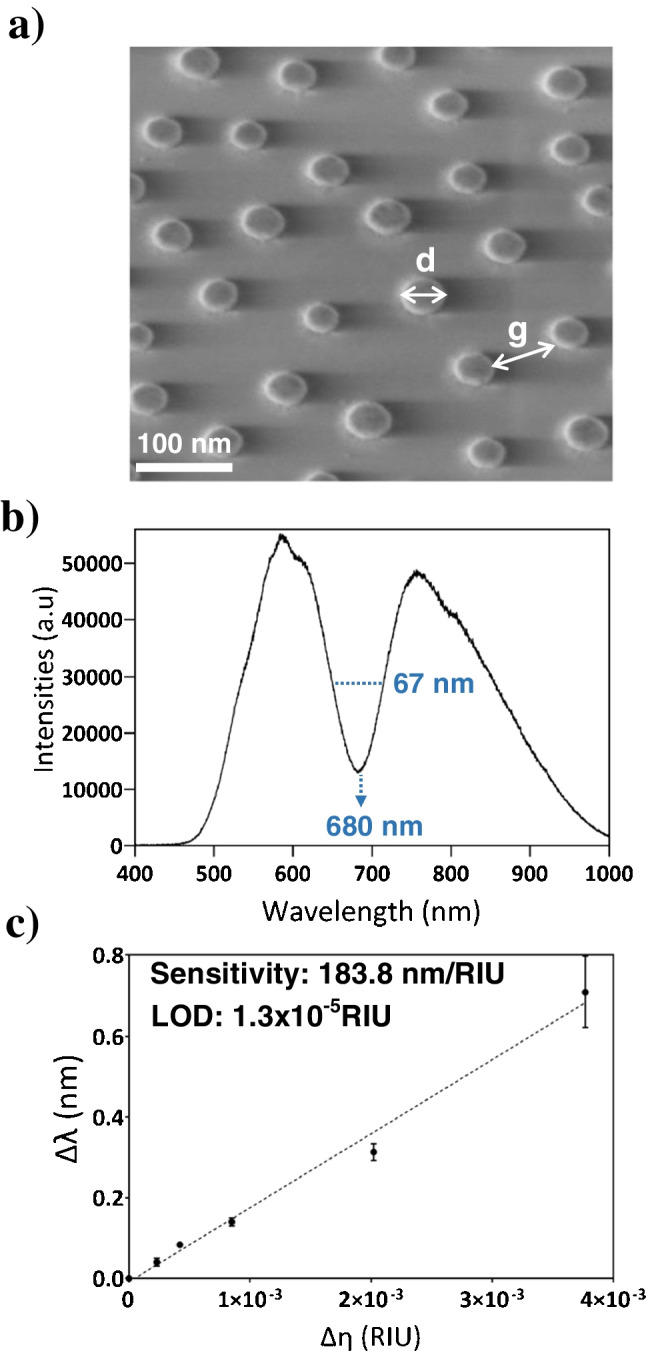
(**a**) Scanning electron microscopy (SEM) image of 80-nm AuNDs fabricated using the HCL method, (**b**) spectrum of the reflected light of the nanoplasmonic sensor showing the LSPR peak around 680 nm, and (**c**) bulk sensitivity calibration obtained with sequential measurements of different HCl concentrations over a Milli-Q water running flow. Each measurement corresponds to the mean ± SD of triplicate measurements

To fabricate our new nanoplasmonic sensors, we employed a previously established procedure based on hole-mask colloidal lithography (HCL) [5, 6, 14]. This technique renders short-ordered arrays of nanostructures with high reproducibility and good precision, it is relatively simple to perform, and allows for large-scale production of sensors at low cost. In addition, the HCL procedure offers the possibility to easily tailor the diameter and the final density of nanostructures by selecting the appropriate material, size, and concentration of colloidal particles. In particular, to produce low-density gold nanodisk arrays, we employed a 0.2% colloidal polystyrene beads solution with 80-nm diameter. The resulting nanoplasmonic sensors were characterized using microscopy techniques to examine the surface features and distribution of gold nanostructures. Scanning electron microscopy (SEM) images showed a short-ordered distribution of nanodisks of approximately 80-nm diameter, as expected, and the density of the nanostructures indeed occupies between 6 and 7% of the glass substrate (Fig. 2a). Three-dimensional (3D) scans of the nanoplasmonic substrate were obtained using atomic force microscopy (AFM), confirming the 20-nm height of the nanostructures and the interparticle average distance of approximately 80 nm (*g* = *d*) (Fig. S3), Additionally, contact angle analysis was conducted to determine the wettability of the sensors and it was compared to bare glass substrates and thin-film gold sensors (Fig. S4). The average contact angles of the nanoplasmonic sensors and the glass substrate were measured at 42.3° and 44.5°, respectively, while the contact angle for a recently cleaned gold surface was determined at 59.2°. This experiment confirmed our hypothesis that a low-density nanoplasmonic array can exhibit the same hydrophilic behaviour than bare glass substrates, being significantly higher than plasmonic metal surfaces. Therefore, since the formation of SLB on glass substrates has been previously demonstrated, we could expect the successful formation of SLB on our AuNDs sensors with an analogue hydrophilic behaviour.

Finally, the 80-nm AuNDs sensors were characterized and evaluated for label-free refractometric sensing. The sensors were mounted into a LSPR device working in prism-coupling configuration by wavelength interrogation. The nanoplasmonic sensors exhibited an intense and narrow resonance peak at around 680 nm, with a narrow bandwidth (full width half maximum, FWHM of 67 nm) (Fig. 2b), as predicted by our simulations (Fig. S1b). By performing a bulk sensitivity study (i.e., measurement of bulk refractive index changes due to sequential introduction of HCl dilutions), the sensitivity of the sensor was determined at 183.8 nm/RIU and the limit of detection was calculated at 1.3 × 10^−5^ RIU (Fig. 2c). Compared to our previous 100-nm AuNDs study [6], this new nanoarchitecture with smaller AuNDs diameters resulted in a slightly lower sensitivity, but an equivalent or superior resolution for the detection of low concentrations, mainly provided by the narrower peak bandwidth. Furthermore, our AuNDs refractometric sensing performance is comparable to the state-of-the-art nanoplasmonic sensors previously reported, ranging between 10^−4^ and 10^−6^ RIU [16–18].

### Formation of a PD-L1-functionalized lipid membrane

To develop a biomimetic sensor for immunotherapy evaluation, the AuNDs sensors were functionalized with an artificial tumor cell membrane, essentially formed by a supported lipid bilayer (SLB) decorated with PD-L1 molecules. The immobilization of PD-L1 molecules on a planar and fluid lipid membrane would allow for the lateral mobility of ligands to maximize the efficiency in the recognition of its corresponding PD1 receptor. For the formation of a functional SLB, two key factors must be considered: the preparation of small unilamellar vesicles (SUV) with an appropriate ratio of stabilizers and functional phospholipids (i.e., carrying a reactive chemical moiety at the head), and the high hydrophilicity of the sensor substrate, which will promote the SUV disruption and re-arrangement into a planar bilayer. As mentioned before, the conventional approach for ensuring the hydrophilic surface is to coat the plasmonic metal with a SiO_2_ layer, decreasing the overall sensitivity of the sensors and increasing the complexity of the nanofabrication procedure. In our case, we have developed a low-density nanostructured sensor with similar hydrophilicity behavior to bare glass, so we expect the successful formation of SLB directly on the nanoplasmonic sensor. Regarding the SUV composition, we selected a 10:1 mixture of POPC and DOPS, in which the POPC serves as stabilizer and the DOPS will provide functional carboxylic (COOH) groups for subsequent anchoring to PD-L1 molecules.

**Fig. 3:**
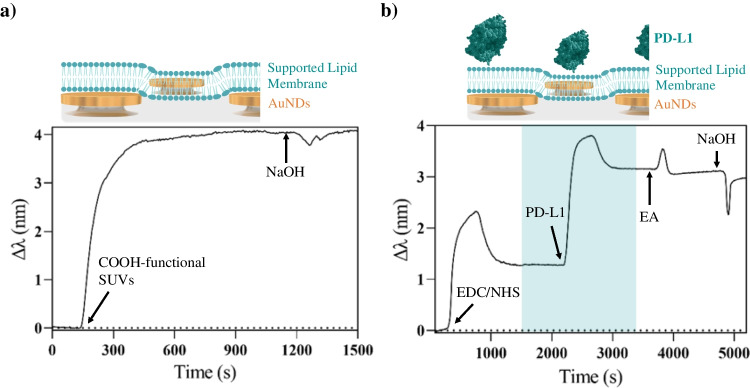
(**a**) LSPR sensorgram showing the formation of a supported lipid membrane (SLB) through the disruption of small unilamellar vesicles (SUVs) followed by a NaOH cleaning step, and (**b**) LSPR sensorgram showing the covalent immobilization of PD-L1 molecules on the SLB via EDC/NHS chemistry. The figure is only for illustrative purposes; elements (nanodisks, lipids, proteins, and antibodies) are not to scale

The colloidal solution of functional SUVs was prepared by membrane extrusion to ensure a maximum diameter of 100 nm, which makes them highly unstable and prone to disrupt upon contact with a hydrophilic substrate. Once ready, the SUV solution was rapidly injected into the LSPR device, and the vesicle disruption and planar bilayer formation were monitored. As can be observed in Fig. 3a, the sensorgram shows a prompt signal increase up to 4 nm, followed by signal stabilization for 5 min. To ensure the robustness and stability of the planar lipid bilayer, we introduced a NaOH solution for 2 min, which did not affect the functionalized signal (i.e., baseline remains unchanged). In the event that SUVs had attached to the sensor surface through electrostatic interactions instead of disrupting, the change of pH would have removed them (as illustrated in Fig. S5), resulting in a decrease of the sensor signal. Our sensorgrams not only exhibit the characteristic shape and peak shift indicative of SLB formation on nanoplasmonic sensors, as described by Jonsson et al. [7], but also demonstrate consistency across multiple measurements, with a coefficient of variation around 6%. Additionally, the successful formation of the SLB is further validated by the stability observed during a pH change experiment using NaOH injection at *t* = 1200 s. The lack of alteration in the sensor signal following the pH change confirms the robustness and stability of the SLB on the nanoplasmonic sensor surface.

After SLB formation, the PD-L1 molecules were immobilized by covalent binding to the carboxylic groups available from the DOPS. We employed a recombinant PD-L1 Fc chimera, as it offers terminal amine (NH_2_) groups in the Fc region that can serve as an anchoring entity to the SLB through EDC/NHS chemistry while ensuring accessibility to the PD-L1 binding sites for PD1 recognition. In Fig. 3b, the sensorgram corresponding to the covalent immobilization process of the PD-L1 is shown. As can be seen, the PD-L1 binding produced a significant signal increase (Δ*λ* ~ 3 nm), which was stable after ethanolamine deactivation and a NaOH cleaning step. The reproducibility and robustness of the complete biofunctionalization process were evaluated over a series of more than 15 sensor chips, with coefficients of variation of 6% for the SLB formation and 12% for the PD-L1 immobilization.

### Highly sensitive analysis and monitoring of PD1/PD-L1 interactions

**Fig. 4:**
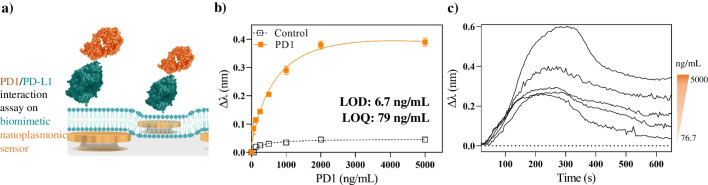
**(a**) Schematic illustration of PD1 binding to PD-L1 ligands anchored to biomimetic nanoplasmonic sensor; (**b**) calibration curve performed for the detection of PD1 receptor, including data for a negative control proteins (square) at different concentrations; and (**c**) representative LSPR sensorgrams obtained for different concentrations of PD1 binding to PD-L1. All the measurements were performed in triplicate for each data point. The figure (**a**) is only for illustrative purposes; elements (nanodisks, lipids, proteins, and antibodies) are not to scale

To demonstrate the applicability of our biomimetic nanoplasmonic sensor, we first addressed the analysis and monitoring of PD-L1 and PD1 immune checkpoint interactions. By using recombinant PD1 receptors, a direct label-free detection assay was optimized in terms of buffer composition and pH (Fig. S6) to ensure maximum recognition efficiency by PD-L1 while avoiding possible non-specific interactions. We exposed the immobilized PD-L1 molecules on the biomimetic interface to a range of PD1 concentrations (0 to 5000 ng/mL) diluted in HEPES buffer (Fig. 4a) and monitored the real-time interaction with our LSPR technology. The acquired sensor signals (Δ*λ*) were plotted against the PD1 concentrations, obtaining a calibration curve exhibiting the typical one-site saturation binding profile (Fig. 4b). Possible non-specific interactions were also evaluated by introducing a negative control sample at different concentrations (C-reactive protein, CRP), which produced negligible signals, confirming that the sensor response is essentially due to the PD1/PD-L1 interactions, and that the lipid membrane efficiently covers the whole sensor surface, preventing from possible non-specific adsorptions. From the calibration curve, the limit of detection (LOD) and limit of quantification (LOQ) were determined at 6.7 ng/mL (0.2 nM) and 79 ng/mL (2.6 nM), respectively. These values demonstrate the excellent sensitivity of our biosensor, achieving LODs up to one order of magnitude better than those previously reported with plasmonic sensors for the analysis of PD1/PD-L1 interaction, obtaining LODs at 0.77 µM [19] and 1.9 µM [20]. The reason could be attributed to the unique biomimetic design of our nanoplasmonic biosensor, which enhances the sensitivity compared to conventional SPR sensors by enabling lateral mobility of the PD-L1. Besides, our LSPR platform also facilitates the direct monitoring and analysis of PD1/PD-L1 sensorgrams (Fig. 4c), which could be used to determine affinity and kinetic parameters of different checkpoint inhibitors or other cell receptor-ligand interactions. Finally, it is worth mentioning that the complete affinity assay can be performed in less than 20 min with a 150 µL sample volume, without requiring any other specific reagent, and with a compact and easy-to-use instrument.

### Assessment of monoclonal antibody (mAb) as PD1 checkpoint inhibitor

**Fig. 5:**
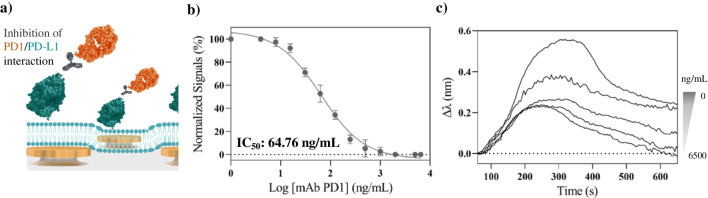
Evaluation of mAb as PD1 checkpoint inhibitor. (**a**) Schematic illustration of the competitive assay performed with an anti-PD1 mAb over the PD-L1-functionalized biosensor, (**b**) dose–response inhibition curve obtained for a fixed concentration of PD1 (2000 ng/mL) incubated in a series of dilutions of anti-PD1 mAb, and (**c**) LSPR sensorgrams obtained for different concentrations of anti-PD1 mAb mixed with PD1 receptors, and their binding to PD-L1. All the measurements were performed in triplicate for each data point. The figure (**a**) is only for illustrative purposes; elements (nanodisks, lipids, proteins, and antibodies) are not to scale

Finally, our biomimetic nanoplasmonic sensor was applied for the evaluation of PD1 checkpoint inhibitors as candidates for cancer immunotherapy. We tested the performance of a monoclonal antibody (mAb) targeting the PD1 receptor for blocking the interaction with the immobilized ligands (PD-L1) (Fig. 5a). The inhibition assay was carried out by incubating a fixed concentration PD1 (2000 ng/mL) with serial dilutions of anti-PD1 mAb (0–6500 ng/mL) for 10 min, and then flowing the mixture onto the biomimetic sensor surface expressing PD-L1 molecules. The mAb binding to the PD1 receptor is expected to hamper the interaction of PD1 with the immobilized ligands, therefore decreasing the sensor response. The dose–response inhibition curve obtained is shown in Fig. 5b, together with representative sensorgrams (Fig. 5c). As expected, increasing concentrations of the mAb produced a decrease of the signal corresponding to PD1/PD-L1 interaction, achieving the complete inhibition of the checkpoint interaction at 2000 ng/mL mAb concentration. We also tested a negative control sample (mAb 6500 ng/mL with no PD1 receptor) that resulted in negligible signal, and a blank sample with PD1 protein and no antibody, showing the highest signal. From the dose–response curve, we determined the half maximum inhibition concentration (IC_50_) for this anti-PD1 mAb at 64.76 ng/mL (0.43 nM).

The IC_50_ value obtained with our biomimetic LSPR sensor is in the same nM range with those derived from conventional methods like SPR, flow cytometry, or other fluorescence-based assays for commercial and non-commercial anti-PD1 mAbs (Table 1). For instance, different works have analyzed and evaluated nivolumab — the most common PD1 inhibitor drug — determining the IC_50_ at 1.3 nM when using fluorescence techniques [21] or at 2.52 nM when using a SPR biosensor [22]. On the other hand, flow cytometry has been widely employed for the study of novel PD1 ICIs, like camrelizumab, cemiplimab, pembrolizumab, or murine mAbs, obtaining values that range from more than 5 to 0.24 nM [23] [24]. Herein, our results perfectly align with an IC_50_ of 0.43 nM for an anti-PD1 mAb, proving the reliability of our innovative biomimetic technology. Additionally, our biosensor stands out being the only technology integrated in a point-of-care device, offering a rapid and label-free analysis, and providing biomimetic conditions that could simulate cell–cell interactions. The high sensitivity of our device would further facilitate the pre-clinical studies by reducing the amount of reagents and cell samples, thereby reducing the associated costs and ultimately potentially lowering the production expenses for the therapies.

**Table 1:** Comparison of PD1 checkpoint inhibitors evaluation results with different analytical techniques

Technique	Checkpoint inhibitor	IC_50_ (nM)	Reference
Biomimetic LSPR sensor	Anti-PD1 mAb	0.43	This work
Fluorescence	Nivolumab	1.3	[21]
SPR sensor	Nivolumab	2.52	[22]
Flow cytometry	Camrelizumab	5.6	[23]
Flow cytometry	Cemiplimab	2.6	[23]
Flow cytometry	Pembrolizumab	0.9	[23]
Flow cytometry	Anti-PD1 mAb	0.24	[24]

## Conclusions

We have demonstrated a label-free, rapid, cost-effective, and user-friendly biomimetic nanoplasmonic sensor technology as a biomedical tool for the study and assessment of checkpoint inhibitors in cancer immunotherapy. Our biosensor utilizes short-ordered low-density arrays of plasmonic nanostructures, which can be fabricated at large scales by colloidal lithography, allowing for the direct on-chip formation of artificial cell membrane scaffolds that mimic the tumor microenvironment and cell receptor mobility. This lipid membrane can be easily functionalized and decorated with specific ligands, such as the PD-L1, to monitor and analyze immune checkpoint interactions with high sensitivity (LOD = 6.7 ng/mL). As a proof-of-concept, we have demonstrated the accuracy of our biomimetic sensor for the evaluation of an anti-PD1 mAb as checkpoint inhibitor, obtaining an IC_50_ value in the low nM range, comparable to the values determined with conventional analytical techniques for similar therapeutic formulations. To our knowledge, our work is the first one demonstrating the application of a nanoplasmonic sensor for the label-free and biomimetic study of PD1/PD-L1 pathway inhibition.

The main novelty of this work lies on the rational design of the nanosensor architecture, providing a unique platform for direct formation of supported lipid bilayers, eliminating the need of SiO_2_ coating, and enhancing the detection sensitivity compared to previously reported technologies. Furthermore, the nanoplasmonic biosensor is integrated in a compact device for decentralized and non-specialized operation, promoting its deployment to biomedical and pharmaceutical research laboratories. Finally, the large versatility of our approach will be key to facilitate the comprehensive and effective screening of different ICIs, the study of novel immune checkpoint targets, or possible tumor resistance mechanisms. Our biomimetic sensor technology could greatly contribute to the research in advanced cancer immunotherapies, improving their efficacy, and accelerating their validation and implementation in clinics.

## Supplementary Information

Below is the link to the electronic supplementary material.

Supplementary file1 (DOCX 3696 KB)
