# Supplementary material for: A novel biomimetic nanoplasmonic sensor for rapid and accurate evaluation of checkpoint inhibitor immunotherapy
Source: Anal Bioanal Chem. 2024 Jun 20;416(30):7295–304. doi: 10.1007/s00216-024-05398-3 (PMC11584438; doi:10.1007/s00216-024-05398-3)
Supplement: Supplementary file 1 — Supplementary file1 (DOCX 3696 KB) [file 216_2024_5398_MOESM1_ESM.docx]

**Supplementary Information**

**A novel biomimetic nanoplasmonic sensor for rapid and accurate
evaluation of checkpoint inhibitor immunotherapy**

Razia Batool, Maria Soler*, Rukmani Singh, Laura M. Lechuga^†^

*Nanobiosensors and Bioanalytical Applications Group (NanoB2A), Catalan Institute of Nanoscience and Nanotechnology (ICN2), CSIC, BIST and CIBER-BBN, 08193 Bellaterra, Barcelona, Spain*

* Corresponding author: [maria.soler@icn2.cat](mailto:maria.soler@icn2.cat)

^†^ CSIC corresponding author: [laura.lechuga@icn2.cat](mailto:laura.lechuga@icn2.cat)

**Table of Contents**

**S1**. Numerical modeling of nanoplasmonic arrays

**S2.** Fabrication of nanoplasmonic sensors

**S3:** Atomic force microscopy (AFM) characterization

**S4**. Contact angle analysis.

**S5**. Examples of failed experiments involving supported lipid bilayer (SLB) formation on different plasmonic surfaces.

**S6.** Optimization of assay parameters for PD1/PD-L1 monitoring

**S1**. **Numerical modeling of nanoplasmonic arrays**


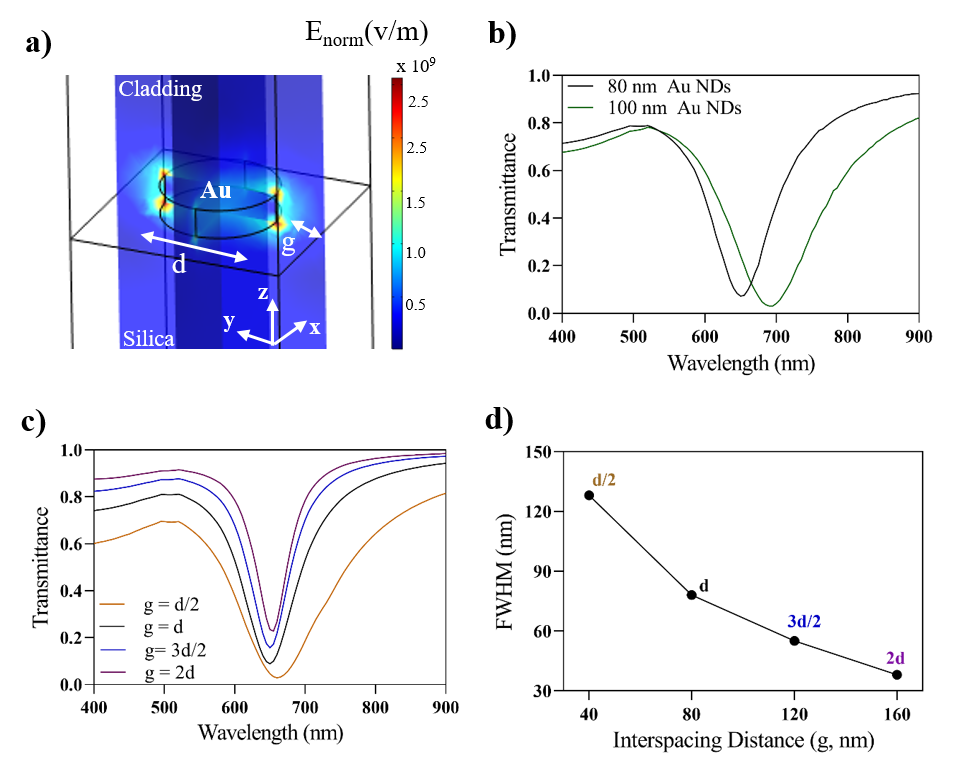


**Fig. S1.** **a)** Electric field distribution in a three-dimensional unit cell of the short-ordered nanoplasmonic arrays; **b)** Transmission spectra of 80 nm AuND (black) and 100 nm AuNDs (green); **c)** Interspacing distance (g) analysis in range of d/2 to 2d for 80 nm AuND (d = diameter); **d)** FWHM values for different interspacing distance between AuNDs.

**S2. Fabrication of nanopasmonic sensor**

The AuNDs nanofabrication protocol, based on Hole-Mask Colloidal Lithography (HCL), is detailed as follows. First, glass substrates (20 x 26 mm No.4, Menzel-Glaser, Germany) underwent a thorough cleaning process. This involved a sequential cleaning with SSD (0.5 % wt), HCl (0.1 M) and mili-Q water, dry with nitrogen (N_2_) stream. Subsequently, the substrates were immersed in a freshly prepared piranha solution (3:1, H_2_SO_4_:H_2_O_2_) for 20 minutes. After rinsing and drying with N_2_ stream, the HCL fabrication was initiated. Before depositing the colloidal sphere beads, a 200 nm-thick layer of 950 K Polymethyl methacrylate (4% PMMA in 4% anisole) was spin-coated (4000 rpm, 3000 r.s^-2^) on piranha-cleaned glass substrates. Following a 5-minute baking process at 155° C temperature, the substrates were subjected to a brief exposure to O_2_ plasma (18s, 75W, 75 mTorr, 40% O_2_-flux) to enhance the hydrophilicity of PMMA-coated substrates. An oppositely charged polyelectrolyte was drop casted onto the PMMA layer to create an adhesive surface. For this purpose, surface was coated with 0.2 wt% PDDA (Sigma-Aldrich, Germany) for 1 min and thoroughly rinsed with mili-Q water and dried with N_2_ stream. Following this, colloidal solutions containing 0.2 wt% of 80 nm diameter of polystyrene beads (Invitrogen, USA) were deposited for 1 min, subsequently rinsing with milli-Q water and drying with N_2_ stream, respectively. The electrostatic repulsion between the colloidal sphere beads and the attractive force between these spheres’ particles and the PDDA layer define a randomly arranged nanoarrays of polystyrene spheres. The hole-mask template was formed by evaporating a 15 nm of titanium (Ti) layer as a sacrificial layer using e-beam evaporator. This sacrificial layer is resistive to O_2_ plasma treatment. Then, colloidal spheres were stripped-out by using blue tape, resulting in the removal of the spheres while retaining a PMMA sacrificial layer with a perforated titanium layer, which acts as a mask here, on the top. The sacrificial PMMA layer beneath the holes was removed by O_2_ plasma etching treatment, applied for 300 s at 75 W, 75 mTorr with 50 % O_2_-flux. The resulting masks were utilized as a deposition mask for the gold nanodisks (AuNDs). Using electron-beam evaporation, a 1.5 nm thick adhesive layer of Ti was initially deposited, followed by a 19 nm layer of Au evaporation. Finally, the remaining mask was removed through a lift-off process through sonication in acetone and Milli-Q water for 1 minute each at room temperature, followed by rinsing with Milli-Q water and drying using a N_2_ stream.


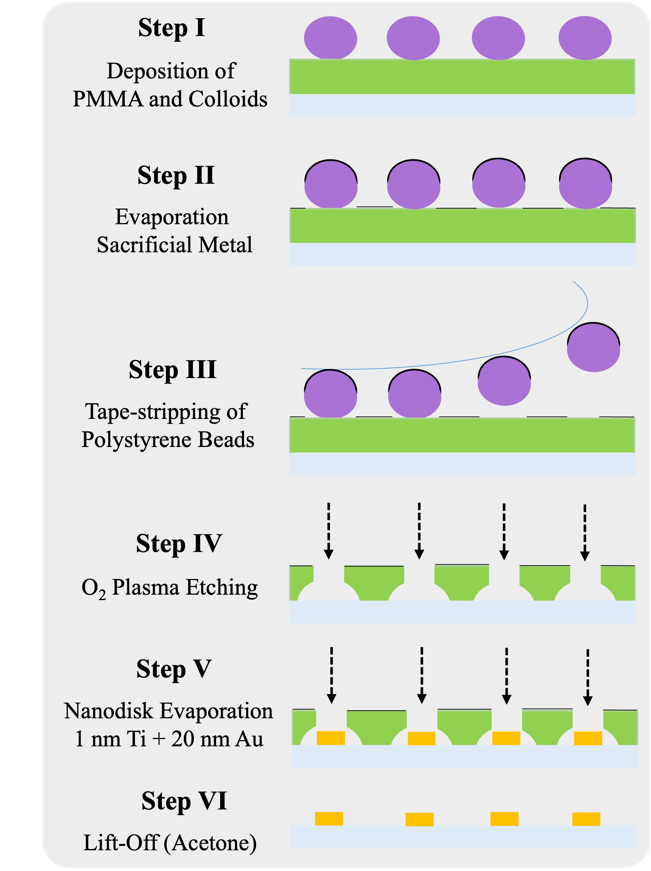


**Fig. S2**. Schematic illustration of the fabrication protocol based on hole-mask colloidal lithography (HCL) technique.

**S3: Atomic force microscopy (AFM) characterization**


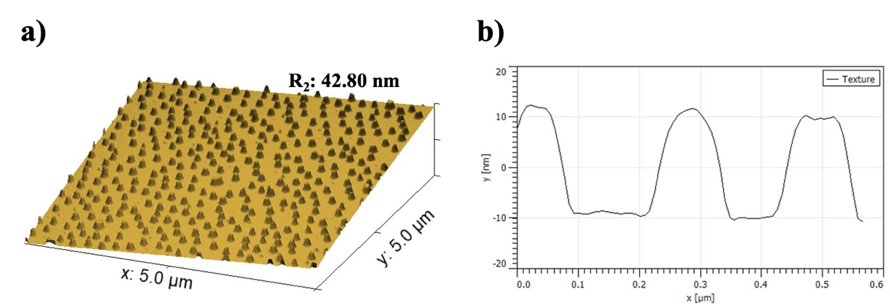


**Fig. S3.** **a)** Atomic force microscopy (AFM) 3D scan of 80 nm AuNDs; **b)** Lateral profile representation of the AFM image.

**S4. Contact angle analysis**


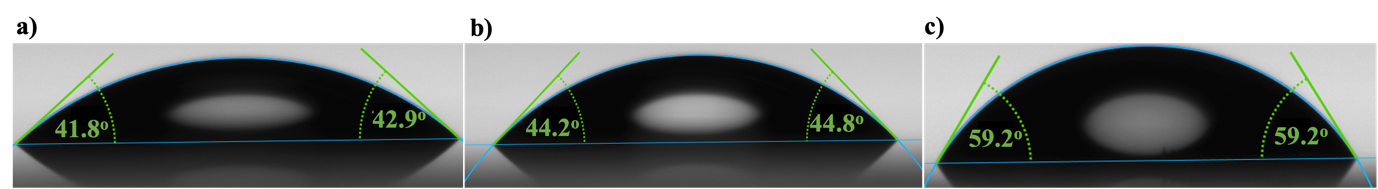


**Fig. S4.** Contact angle analysis of: **a)** AuNDs sensor; **b)** SiO_2_ substrate; **c)** Au thin film.

**S5. Examples of failed experiments involving supported lipid bilayer (SLB) formation on different plasmonic surfaces**


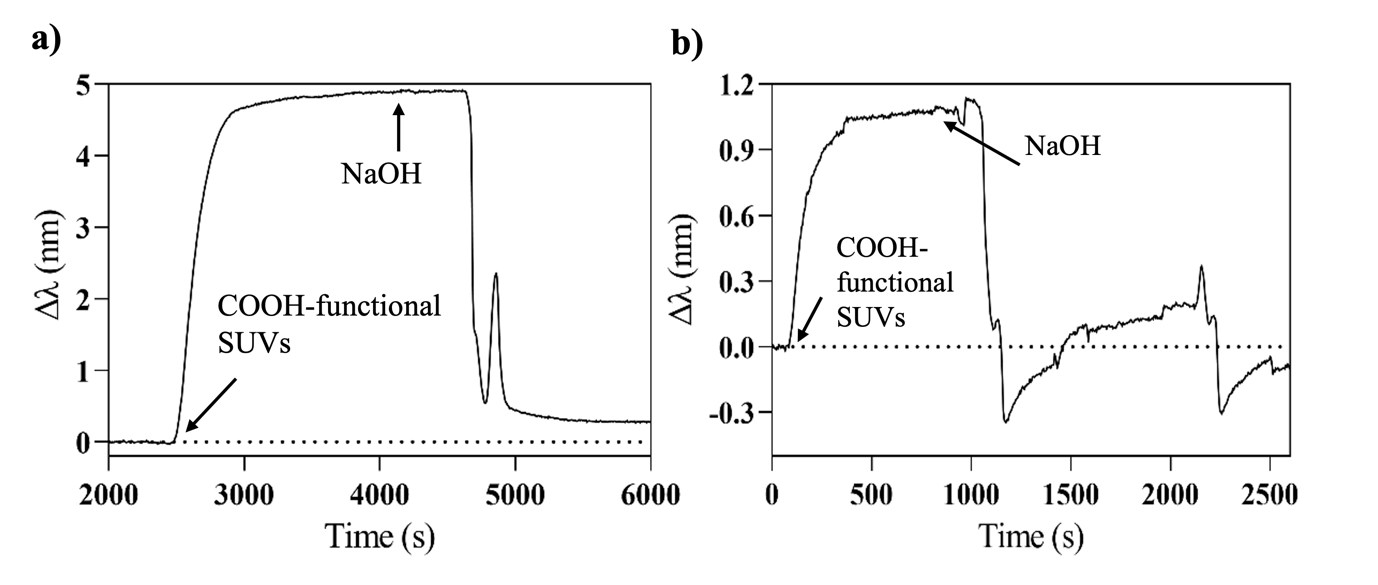


**Fig. S5.** LSPR sensorgram showing the failed formation of a supported lipid membrane (SLB) through the disruption of small unilamellar vesicles (SUVs) followed by a NaOH cleaning step on different plasmonic ssensors; **a)** Gold thin film, and **b)** AuNDs of 100 nm diameter.

**S6. Optimization of assay parameters for PD1/PD-L1 monitoring**

The purpose of this study was to analytically assess and optimize the influence of buffer composition and pH on the binding kinetics and specificity of PD1/PD-L1 interactions. We flowed a constant concentration of 2000 μg/mL PD1 over 50 μg/mL PD-L1 covalently immobilized on a biomimetic scaffold formed on a low-density gold nanostructured substrate, enabling systematic investigation of PD1/PD-L1 interactions across diverse range of pH levels (5 to 9) and different buffers (**Fig. S6a)**. Additionally, we evaluated the non-specific interactions to measure the assay specificity under each buffer condition (**Fig. S6b)**. Taking into account the maximum detection signal and minimum non-specific binding, best results were obtained with HEPES buffer at pH 6.5, which was selected for subsequent experiments.


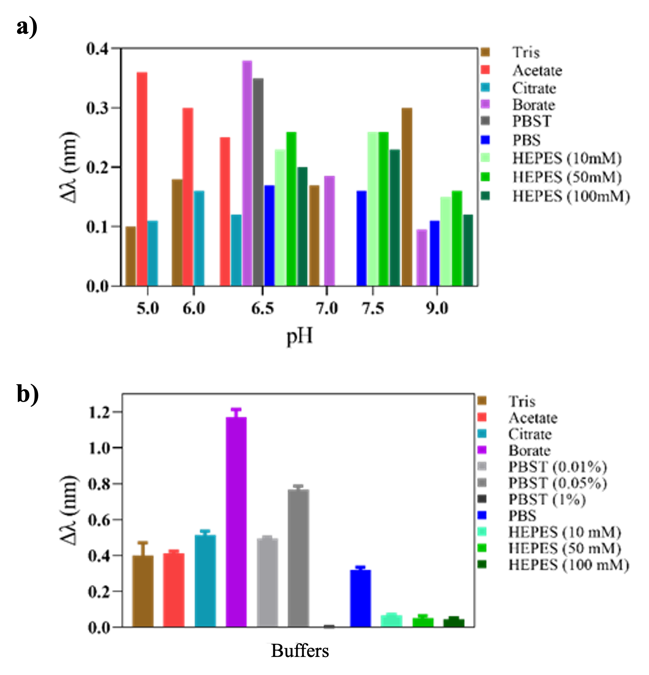


**Fig. S6.** **a)** pH and buffers optimizations for PD1/PD-L1 high affinity interactions; **b)** Assessment of non-specific interactions in different buffers.
